# Esencia de Calisaya

**Published:** 1893-11

**Authors:** 


### ESENCIA DE CALISAYA.

Since this preparation was first put upon the market by the Mosquera-Julia Food Co., of Detroit, Mich., it has grown rapidly in favor as a tonic, stomachic, and to a lesser extent as an anti-periodic. There are few cases in the convalescent stage to which it is not applicable, especially if anorexia, weakness, and physical and mental depression are to be overcome.

It is a well-known fact, moreover, that the combined alkaloids of the cinchona bark are much more effective as a tonic than any one of the same taken singly. They are to be preferred in many instances as an anti-periodic, particularly where the periodicity of the attack has been in some degree mitigated. It is for this reason that the East India Government now provides its officials with what is termed "Cinchona Febrifuge"—which is but a combination of cinchona alkaloids—in preference to quinine. While cases are encountered where quinine seems practicably indispensable, there are few that will not yield, and more satisfactorily, to a combination of cinchona alkaloids if persisted in.—Esencia de Calisaya and Cinchona Febrifuge are practically identical, save that the former is a fluid medicament, the latter a powder.

It is for the foregoing reasons and to meet the demand of a large class of medical men, that Esencia de Calisaya has been put upon the market; recently, too, its scope has been increased somewhat, in that it is employed and highly recommended as a tonic in neurasthenia and other forms of disease that are the result of, or connected with, general physical and mental depression. *Apropos* of this, Dr. Wyatt Wingrave, Surgeon at the London Throat and Ear Hospital, remarks:

"I have great pleasure in expressing my appreciation of Esencia de Calisaya. It is an ideal 'Pick-me-up' and general tonic. It is slightly stimulant to the gastric mucous membrane, and is particularly useful in atonic dyspepsia. *In the alcohol habit, it satisfactorily neutralizes the craving for spirits,* and will be found of great service in treating this distressing disease."

Other physicians have expressed themselves in like manner, asserting Esencia de Calisaya as a valuable adjunct in the treatment of the liquor habit. It is objected by some that the preparation is too sweet, while others assert the precise contrary. Such a fault, if fault it be, is individual, and consequently cannot be corrected in the process of manufacture where a medicament is applicable to so large a class of diseases, to so extensive a *clientele* as is thus provided.

